# β-1,3-glucan modifying enzymes in *Aspergillus fumigatus*

**DOI:** 10.3389/fmicb.2013.00081

**Published:** 2013-04-17

**Authors:** Isabelle Mouyna, Lukas Hartl, Jean-Paul Latgé

**Affiliations:** Unité des Aspergillus, Département de Parasitologie et Mycologie, Institut PasteurParis, France

**Keywords:** *Aspergillus fumigatus*, cell wall, β-1,3-glucan, β-1,3-glucan modifying enzyme, endo-β-1,3-glucanase, exo-β-1,3-glucanase

## Abstract

In *Aspergillus fumigatus* like in other filamentous ascomycetes, β-1,3-glucan constitutes a prominent cell wall component being responsible for rigidity of the cell wall structure. In filamentous fungi, softening of the cell wall is absolutely required during conidial germination and hyphal branching. Because of the central structure of β-1,3-glucans, it is expected that β-1,3-glucanases play a major role in cell wall softening. Based on *in silico* and experimental data, this review gives an overview of β-1,3-glucan modifying enzymes in *A. fumigatus* genome and their putative role during morphogenesis.

## Introduction

The cell wall of *Aspergillus fumigatus* is predominantly composed of polysaccharides. The central fibrillar core of the cell wall is composed of a polymer of β-1,3-glucan which is a branched glucan with 4% β-1,6 branch points, to which the chitin, the galactomannan and β-1,3-1,4-glucan are covalently bound (Fontaine et al., 2000). All these polysaccharides are specific of fungal cells, and constitute good antifungal target.

**Figure 1:**
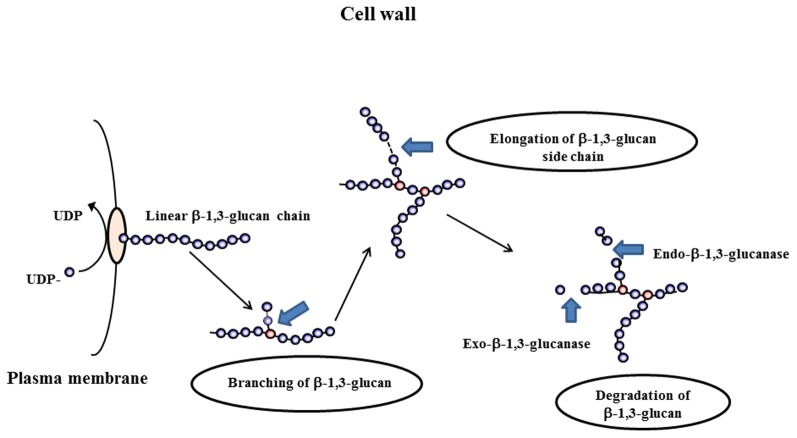
**Differents steps occurring during the remodeling process of the cell wall: branching of β(1–3)glucan, elongation and degradation of β(1–3)glucan chains**.

β-1,3-glucans of *A. fumigatus* are synthesized by a plasma membrane-bound glucan synthase complex, which uses UDP-glucose as a substrate and extrudes linear β-1,3-glucan chains through the membrane into the periplasmic space (Beauvais et al., 1993, 2001). Upon arrival in the cell wall space, newly synthesized polysaccharides are remodeled in order to be incorporated in the pre-existing cell wall. An example of the modifications that occur is given in Figure 1. Steps involved in the remodeling process are structural modifications of the linear glucan chains, produced by the glucan synthase complex, followed by branching, elongation, and degradation of the β-1,3-glucan of the cell wall. Morphogenetic events like conidial swelling (the isodiametral growth phase of germination) germ tube emergence and the production of lateral hypha during mycelial growth require that the cell wall loses its rigidity to allow the emergence of an “additional cell.” Two different biochemical events have been suggested to be associated with germination: the first one is an increased intracellular osmotic pressure (D'Enfert, 1997) and the second one is the softening of the existing cell wall by glycosylhydrolases.

Osmotic pressure is high in resting conidia due to their high content of trehalose and polyol. The molecules are, however, fully degraded during the swelling of the conidium which suggests the occurrence of another biochemical structural modification event to facilitate the remodeling of the cell wall during germination. Glycosylhydrolases are the candidates of choice for these cell wall modifications.

Here we present a survey of potential endo-β-1,3-glucanases and exo-β-1,3-glucanases found in the *A. fumigatus* genome as well as the already identified glucanases and glucanosyltransferase activities described in *A. fumigatus* (Table 1). A survey of β-1,3-glucanases was undertaken in the genomes of *A. fumigatus* strain Af293 (Nierman et al., 2005) using the cadre genome (http://www.cadre-genomes.org.uk/Aspergillus_fumigatus) and the Carbohydrate-Active enZYme (CAZy) Database (http://www.cazy.org/).

**Table 1:** **AFUA-number of *A. fumigatus* genes from Af293 strain (****http://www.cadre-genomes.org.uk/Aspergillus_fumigatus****) and characteristics of modifying cell wall β-1,3-glucanases of *A. fumigatus.***

**Genes**		**Family**	**Size (aa)**	**SP**	**pI**	**Expressed in vegetative mycelium**
**EXOβ(1–3)GLUCANASE**						
*EXG1*	AFUA_1G03600	GH5	416	+	4.3	+
*EXG2*	AFUA_6G09250	GH5	833	−	7.9	+
*EXG3*	AFUA_7G05610	GH5	470	−	6.1	−
*EXG4*	AFUA_2G09350	GH5	396	+	4.8	+
*EXG5*	AFUA_6G11980	GH55	822	−	4.5	−
*EXG6*	AFUA_6G13270 ExoGI	GH55	804	+	4.8	−
*EXG7*	AFUA_3G07520	GH55	809	+	4.9	−
*EXG8*	AFUA_1G14450	GH55	947	+	5	+
*EXG9*	AFUA_2G00430	GH55	1005	−	5	−
*EXG10*	AFUA_4G03350	GH55	779	+	5.7	+
**ENDOβ (1–3)GLUCANASE**						
*ENGL1*	AFUA_1G04260	GH81	974	+	5.8	+
*ENG2*	AFUA_2G14360	GH16	652	+	4	+
*ENG3*	AFUA_1G05290	GH16	356	+	5	−
*ENG4*	AFUA_5G02280	GH16	391	−	5.6	+
*ENG5*	AFUA_4G13360	GH16	378	−	6.1	−
*ENG6*	AFUA_6G14540	GH16	285	+	4.6	−
*ENG7*	AFUA_3G03080	GH16	285	+	4.9	−
*ENG8*	AFUA_5G14030	GH16	456	+	5.5	−
**OTHERS EXO-β–GLUCANASE**						
*EXG12*	AFUA_1G05770 ExoGII	GH3	873	+	4.8	+
*EXG13*	AFUA_7G06140	GH3	739	+	5.8	+
*EXG14*	AFUA_7G00240	GH3	767	−	8.4	+
*EXG15*	AFUA_1G17410	GH3	769	+	5	+
*EXG16*	AFUA_8G02100	GH3	806	+	5.3	−
*EXG17*	AFUA_6G14490	GH3	829	−	6.1	−
*EXG18*	AFUA_5G07190	GH3	838	−	5.1	+
*EXG19*	AFUA_6G11910	GH3	856	−	5.9	−
*EXG20*	AFUA_6G08700	GH3	888	+	5.7	+
*EXG21*	AFUA_6G03570	GH3	1033	−	5	+
**BRANCHING ENZYME**						
*BGT1*	AFUA_1G11460	GH17	305	+	4.8	+
*BGT2*	AFUA_3G00270	GH17	446	+	4.7	+
*BGT3*	AFUA_5G08780	GH17	688	+	4.7	+
*SCW4*	AFUA_6G12380	GH17	369	+	7	+
*SCW11*	AFUA_8G05610	GH17				+
**ELONGATION ENZYME**						
*GEL1*	AFUA_2G01170	GH72	452	+	4.7	+
*GEL2*	AFUA_6G11390	GH72	475	+	4.4	+
*GEL3*	AFUA_2G12850	GH72	544	+	4.4	−
*GEL4*	AFUA_2G05340	GH72	548	+	4.6	+
*GEL5*	AFUA_8G02130	GH72	537	+	4.2	+
*GEL6*	AFUA_3G13200	GH72	460	+	4.4	−
*GEL7*	AFUA_6G12410	GH72	541	+	4.7	−
**CROSS LINKING ENZYME**						
*CRH1*	AFUA_6G03230	GH16	357	+	4	+
*CRH2*	AFUA_2G03120	GH16	443	+	4.4	+
*CRH3*	AFUA_3G09250	GH16	363	+	5	+
*CRH4*	AFUA_6G08510	GH16	450	+	4.8	+
*CRH5*	AFUA_1G16190	GH16	395	+	4.4	+

SP, Signal peptide; +, Genes expressed and −, Genes not expressed, in either liquid or aerial growth (Gibbons et al., 2012).

## Are β–glucanase involved in the cell wall modification?

β-1,3-Glucan hydrolyzing enzymes can be divided into exo-β-1,3-glucanases and endo-β-1,3-glucanases. Endo-β-1,3-glucanase activities cleave inside a glucan chain in a more or less random fashion, while the exo-β-1,3-glucanase activities release glucose residues from the non-reducing end (Figure 1). Theoretically, the cleavage of long chains of β-1,3-glucan should cause a softening of the cell wall. Therefore, we expect this type of enzyme to be involved in cell wall remodeling that occur during the swelling and germination of conidia or the branching of hyphae.

In order to identify all the putative β-1,3-glucanases in the *A. fumigatus* genome sequenced strain Af293 (Nierman et al., 2005), we used the classification of the Carbohydrates enZYmes database [CAZy database (http://www.cazy.org/)]. This database describes the families of enzymes with structurally related catalytic and carbohydrate binding modules (CBM) that degrade, modify or create glycosidic bonds. In this classification, we selected and further investigated the families which could contain putative β-1,3-glucanases and each potential endo/exo β-1,3-glucanase identified in the *A. fumigatus* genome was used as a query for BLAST searches to identify possible homologs using cadre genome (http://www.cadre-genomes.org.uk/Aspergillus_fumigatus). This search has led to the identification of twenty nine potential exo-β-1,3-glucanases and endo-β-1,3-glucanases that belong to the CAZy GH families 3, 5, 16, 55, and 81 (Table 1). The expression of these different genes in mycelial growth either in liquid or aerial conditions (Gibbons et al., 2012) has been shown in Table 1. The molecular characteristics and the phylogenetic tree of each of the proteins belonging to these families have been investigated.

### Exo-β-1,3-glucanase families

**Figure 2:**
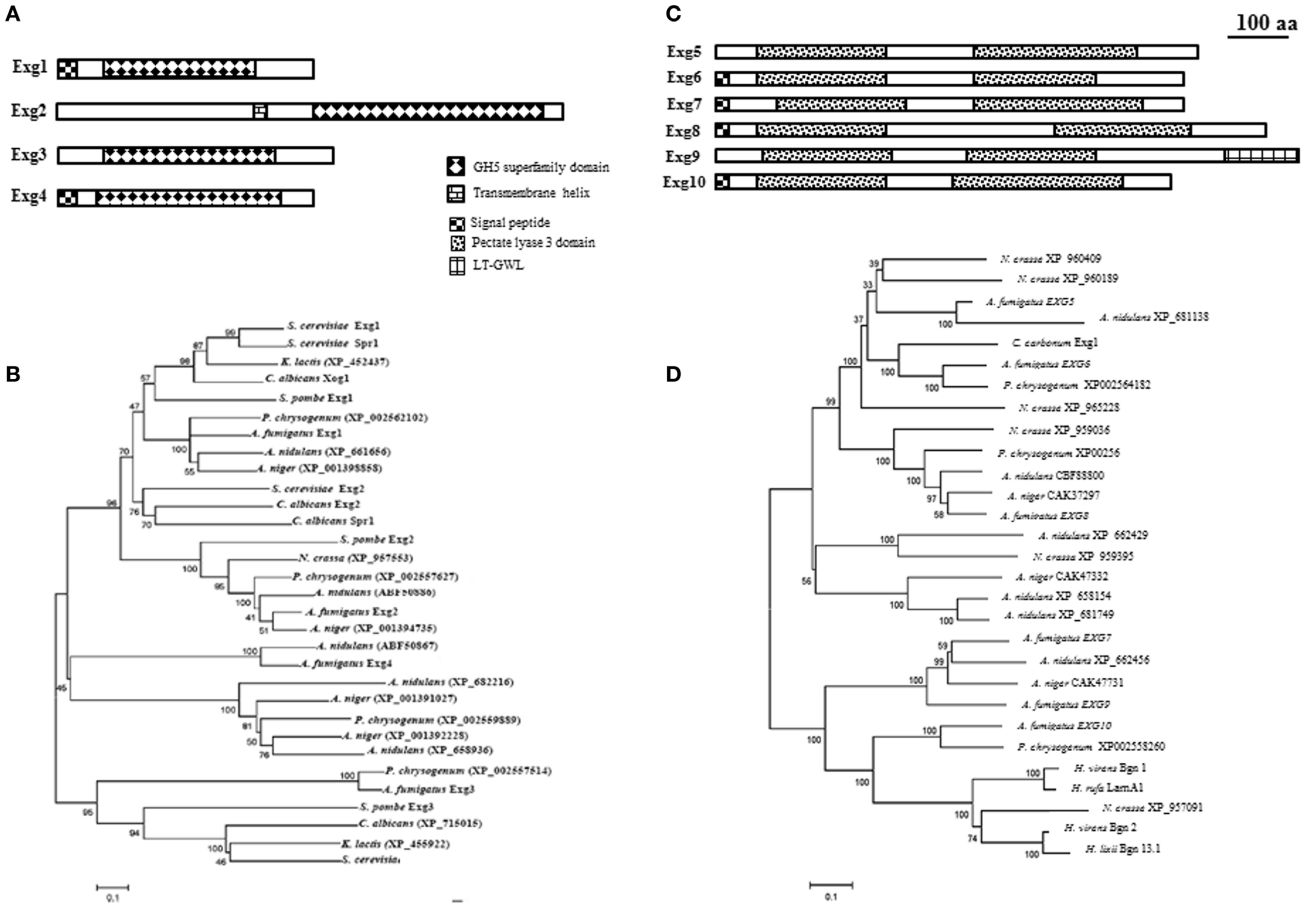
**Domain organization and phylogeny of *A. fumigatus* exo β-1,3-glucanases.** Protein domains identified using SignalP 3.0, big-PI, TMHMM v. 2.0, and CD search (100 aa: protein length proportional to 100 amino acids). Phylogenetic trees of *A. fumigatus* were built by Neighbor Joining. Numbers next to nodes represent bootstrap values calculated using 1000 repetitions. *A. fumigatus* proteins were aligned with orthologs identified in the filamentous fungi *Aspergillus nidulans, Aspergillus niger, Penicillium chrysogenum, Neurospora crassa, Magnaporthe grisea*, and the yeasts *Saccharomyces cerevisiae, Schizosaccharomyces pombe, Kluyveromyces lactis*, and *Candida albicans*. **(A)** GH5 family domain organization. **(B)** Phylogeny of GH5 family, **(C)** GH55 family domain organization, and **(D)** Phylogeny of GH55 glucanases additionally contains sequences of *Cochliobolus carbonum EXG1* (Schaeffer et al., 1994), *Hypocrea rufa* LamA1 (Nobe et al., 2004), *Hypocrea virens* Bgn1 and Bgn2 (Kim et al., 2002), and *Hypocrea lixii* Bgn13.1 (De la Cruz et al., 1995).

In the CAZy database, family GH5 represent a group of hydrolases with different substrate specificities such as for example exo β-1,3-glucanases, endo β-1,4-glucanases, endo β-1,6-glucanases. Four proteins belonging to GH5 family in *A. fumigatus* have been identified in the genome (AfExg1p = AFUA_1G03600, AfExg2p = AFUA_6G09250, AfExg3p = AFUA_7G05610, and AfExg4p = AFUA_2G09350). The characteristics of these proteins are presented in Figure 2A and Table 1.

AfExg1p and AfExg4p have a signal peptide in contrast to the others AfExg2p and AfExg3p. The phylogenetic tree of GH5 proteins is presented in Figure 2B. Recently the respective orthologs in *S. pombe* have been described (Duenas-Santero et al., 2010). SpExg1p is secreted, SpExg2p is bound to the membrane via a GPI anchor and SpExg3p is a cytoplasmic protein. Interestingly, in *S. cerevisiae* and *C. albicans*, it has been shown that Exg1p and Xog1p are able to degrade β-1,3-glucan (Vazquez de Aldana et al., 1991; Chambers et al., 1993) and display exo β-1,3-glucanase activities but Suzuki et al. (2001) showed also that the recombinant ScExg1p was able to cleave also β-1,6-glucan. SpExg1 and SpExg3 are only acting on β-1,6-glucans, while no activity was detected for SpExg2 (Duenas-Santero et al., 2010). In *S. cerevisiae*, the third ortholog *SSG1* has been shown to code for a sporulation specific exo-β-1,3-glucanase activity (San Segundo et al., 1993). In *S. cerevisiae*, the deletion of *SSG1* showed a significant delay in the appearance of mature asci (San Segundo et al., 1993). In contrast, no growth phenotype for the single *exg1* and *exg2* deletion strains of *S. cerevisiae* has been observed (Larriba et al., 1995). Moreover, the triple *ssg1 exg1 exg2* mutants behave like the simple *ssg1* mutant. In *S. pombe*, the phenotype of the triple *exg* mutants behave like the parental strain (Duenas-Santero et al., 2010). None of the *EXG1–4* gene has been deleted or otherwise characterized in *A. fumigatus*.

In the CAZy database, **family GH55** includes exo-β-1,3-glucanases (EC 3.2.1.58) and endo-β-1,3-glucanases (EC 3.2.1.39). In *A. fumigatus*, six proteins belonging to this family are present: AfExg5p = AFUA_6G11980, AfExg6p = AFUA_6G13270, AfExg7p = AFUA_3G07520, AfExg8p = AFUA_1G14450, AfExg9p = AFUA_2G00430, AfExg10 = AFUA_4G03350, (Table 1).

It has been shown that Af*EXG6* encodes a 82 kDa exo β-1,3-glucanase enzyme named ExoGI which hydrolyze exclusively a β-1,3-glucan chain with a minimal substrate size of four glucose residues (Fontaine et al., 1997a). The characteristics of the *A. fumigatus* proteins are shown in Figure 2C and Table 1. AfExg6p, AfExg7p, AfExg8p and AfExg10p feature a signal peptide, while AfExg5p and AfExg9p are intracellular proteins. AfExg9p features an additional LT_GEWL (Lytic Transglycosylase and Goose Egg White Lysozyme) domain. The exoglucanases AfExg5p to AfExg10p all feature two domains with similarity to the pectate lyase 3 domain. GH55 exoglucanases are not found in yeasts like *S. cerevisiae, C. albicans* and *S. pombe*. The protein sequences of the orthologous genes in *Cochliobolus carbonum* Exg1 (Schaeffer et al., 1994), *Hypocrea rufa* LamA1 (Nobe et al., 2004), *Hypocrea virens* Bgn1 and Bgn2 (Kim et al., 2002), and *Hypocrea lixii* Bgn13.1 (De la Cruz et al., 1995) were added to the alignment of the GH55 family to construct a phylogenetic tree (Figure 2D). It has been shown that *C. carbonum* Exg1p and *H. rufa* LamA1p encode for specific exo-β-1,3-glucanases (Nobe et al., 2004) in contrast to Bgn1p, Bgn2p and Bgn13.1p which encode for specific endo-β-1,3-glucanases (De la Cruz et al., 1995; Kim et al., 2002). To date, the biological function of the *A. fumigatus* GH55 glycosyl-hydrolases is unknown since none of these genes have been deleted yet.

### Endo-β-1,3-glucanase families

The specificity of the members of the **family GH16** in the CAZy database is quite wide since members of this GH family can act as xyloglucan xyloglucosyltransferases (EC 2.4.1.207), keratan-sulfate endo-1,4-β-galactosidases (EC 3.2.1.103), endo-1,3-β-glucanase (EC 3.2.1.39), endo-1,3(4)-β-glucanase (EC 3.2.1.6); licheninase (EC 3.2.1.73), β-agarase (EC 3.2.1.81), κ-carrageenase (EC 3.2.1.83), or xyloglucanase (EC 3.2.1.151). The GH16 superfamily has been divided into nine sub-families based on a phylogenetic analysis of conserved domains of members of this superfamily (cd00413; http://www.ncbi.nlm.nih.gov/Structure/cdd/cddsrv.cgi?uid=29534): GH16_lichenases (cd02175), GH16_XETs (cd02176), GH16_kappa_carrageenases (cd02177), GH16_beta_agarases (cd02178), GH16_beta_GRPs (cd02179), GH16_laminarinases (cd02180), GH16_MLG1_glucanases (mixed-linked glucanases, cd02181), GH16_laminarinase_like proteins (cd02182), and GH16_GPI_glucanosyltransferases (cd02183).

**Figure 3:**
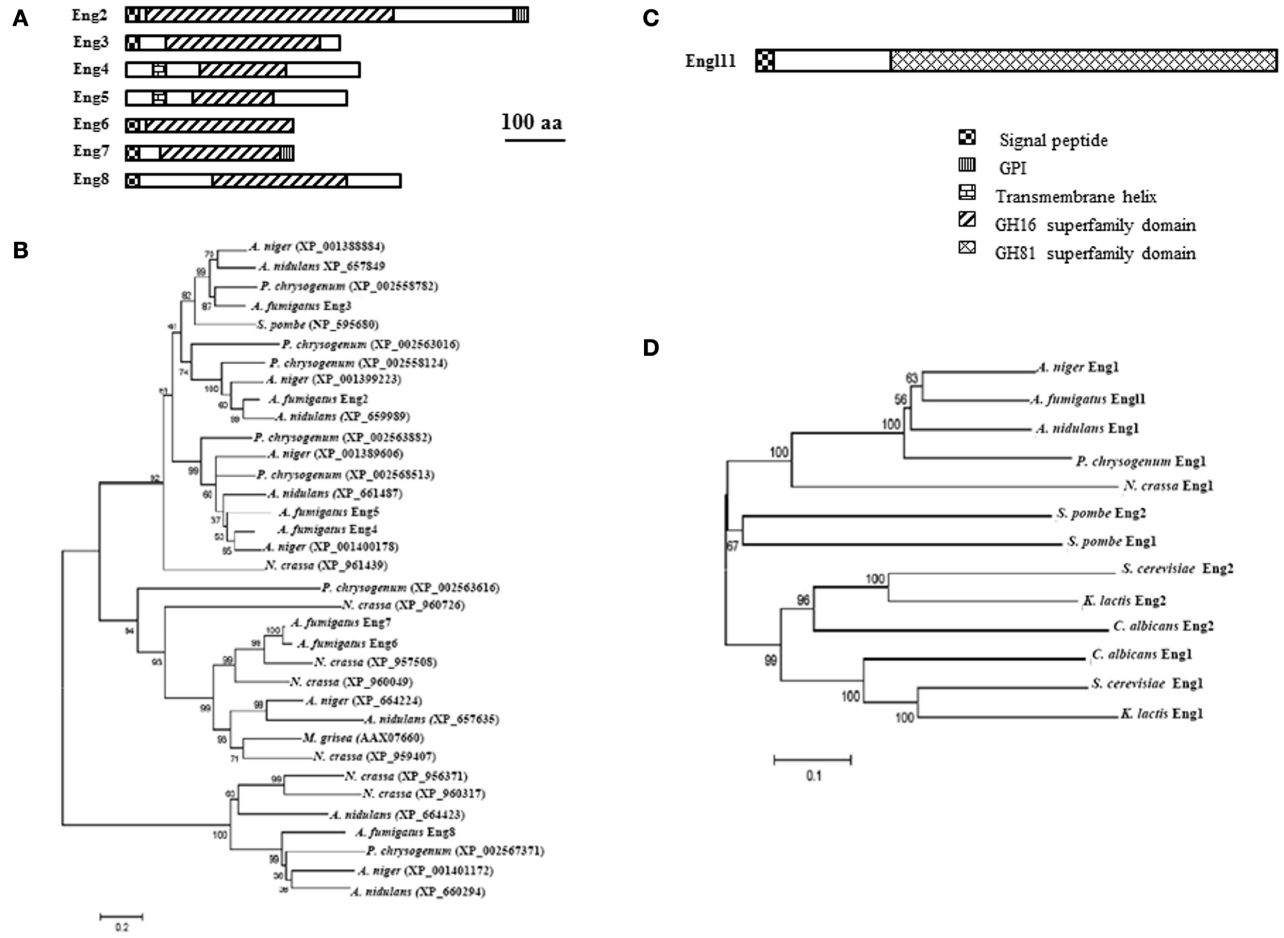
**Domain organization and phylogenetic tree of *A. fumigatus* endo-β-1,3-glucanases.** Protein domains identified using SignalP 3.0, big-PI, TMHMM v. 2.0, and CD search (100 aa: protein length proportional to 100 amino acids). **(A)** GH16 family domain organization and **(B)** phylogeny of GH16 family **(C)** GH81 family domain organization and **(D)** Phylogenetic tree of GH81 family.

In *A. fumigatus*, seven members Af*ENG2* to Af*ENG8* are present in the genome (Table 1, Figure 3A). While AfEng2p to AfEng5p belong to the subfamily GH16_MLG1_glucanases, and AfEng6p and AfEng7p to the subfamily GH16_laminarinase_like proteins, AfEng8p was not assigned to a GH16 subfamily. The cluster of GH16_MLG1_glucanases from AfEng2p to AfEng5p contains four proteins which all share the same catalytic region but aside from that feature different protein domains. AfEng2p and AfEng3p both exhibit an *N*-terminal signal peptide but only AfEng2p, which is considerably larger than its three homologs, holds a GPI signal sequence. AfEng4p and AfEng5p on the other hand are not predicted to contain a signal peptide but both feature a transmembrane helix, suggesting a localization at the plasma membrane with the active domain pointing toward the cell wall. So while these proteins vary in terms of protein domains all four of them might still be localized close to the cell wall, either by being secreted or by being bound to the plasma membrane by a GPI anchor or a transmembrane domain.

It has been shown that Af*ENG2* encode for a endo β-1,3-glucanase protein (Hartl et al., 2011). These enzyme acts on β-1,3-glucans and lichenan but no degradation were observed with β(1–6)substrate. Eng2p preferentially acts on soluble polymers like laminarin and shorter β-1,3- linked oligosaccharides and have a slight transferase activity with substrates of low degree of polymerization (DP4 and DP6). The *eng2* mutant is similar to the parental strain suggesting that AfEng2p alone does not play a morphogenetic role (Hartl et al., 2011). The two GH16_laminarinase_like proteins AfEng6p and AfEng7p exhibit the same protein length and almost identical molecular weights. Both feature a signal peptide followed by the catalytic domain. AfEng7p additionally features a *C*-terminal GPI signal sequence. While the bacterial members of this sub-family often contain a CBM such as CBM6 (Hong et al., 2002) or CBM13 (ricin-type β-trefoil) (Asano et al., 2002; Ferrer, 2006; Shi et al., 2010), no such CBMs were identified in AfEng*6*p and AfEng*7*p.

Although AfEng8p could not be assigned to the licheninase subfamily with high significance, part of the GH16 conserved domain of AfEng8p showed some strong homology to GH16_lichenases (Planas, 2000), which have been shown to be essential for the degradation of extracellular lichenan in *Bacillus subtilis* (Wolf et al., 1995). The fact that AfEng8p also features a signal peptide suggests a similar role in *A. fumigatus*.

In the GH16 family, three clades were identified (Figure 3B): the predicted GH16_MLG1_glucanases (top), GH16_laminarinase_like proteins (middle), and the probable lichenases (bottom). *S. pombe* is the only yeast that possesses a GH16 glucanase (NP_595680). In the filamentous fungi the number of GH16 glucanases ranges from one in *M. grisea* (AAX07660) to eight in *A. fumigatus*. When looking at fungi from the same genus (*A. fumigatus, A. nidulans*, and *A*. *niger*), the total number as well as the number of enzymes present in each clade varies. Because of their endoglucanase activity, multiple deletion of the *eng* gene is ongoing using the beta six rec cassette allowing repetitive round of gene deletion after excision of the resistance marker (Hartmann et al., 2010).

Only endo-β-1,3-glucanase (EC 3.2.1.39) belong to **the GH81 family**. A single member of this family has been found in the *A. fumigatus* genome. It is a glycosylated 74 kDa endo-β-1,3-glucanases protein that was isolated from *A. fumigatus* autolysate (supernatant of the broken mycelium grown 3 days at 37°C) (Fontaine et al., 1997b). This enzyme represents 10–15% of the total β-1,3-glucanase activity found in the *A. fumigatus* cell wall autolysate (supernatant of the cell wall extract incubated 3 days at 37°C). The enzyme recognized at least five glucose units linked by a β-1,3-bond. The gene encoding this activity has been cloned and named *ENGL1* (AFUA_1G04260) (Mouyna et al., 2002). AfEngl1p has an *N*-terminal signal peptide and a large GH81 domain responsible for the endo-1,3-glucanase activity (Figure 3C). In contrast to *A. fumigatus*, two orthologs are present in the *S. serevisiaie, S. pombe*, and *C. albicans* genome. The phylogenetic tree of this GH81 family is presented in Figure 3D. Like for AfEngl1p, it has been shown that SpEng1p, SpEng2p (Martin-Cuadrado et al., 2008a) as well as ScEng1p (Baladron et al., 2002) exclusively hydrolyze linear β-1,3-glucan chains and display the same enzymatic activity as AfEngl1p. Moreover the four conserved aspartic and glutamic acid residues necessary for enzymatic activity has been determined by site-directed mutagenesis in ScEng2p corresponding to D518, D588, E609, and E613 (Martin-Cuadrado et al., 2008a).

In *S. pombe*, Martin-Cuadrado et al. (2003) showed that SpEng1p encodes protein with detectable endo β-1,3-glucanase activity whose deletion interfere in cell separation, because cells fails to degrade the primary septum that separates the two sisters cells. SpEng1p localizes to the septum region at the time of cell separation. SpEng2p also exhibits endo-β-1,3- glucanase activity but mutant Δ*eng2* do not have a cell separation defect. Δ*eng2* of *S. pombe* on the other hand is involved in ascus wall degradation after sporulation (Encinar del Dedo et al., 2009). Esteban et al. (2005) showed in *C. albicans* that CaEng1p is also involved in cell separation. Similar results has been obtained in *S. cerevisiae* (Baladron et al., 2002) where *eng*1 deletion mutants show defects in cell separation. In contrast, the lack of phenotype for the Δ*engl1* mutant suggests that this endo-β-1,3-glucanase activity does not play a morphogenetic role in *A. fumigatus*.

It has been shown that SpEng1p features a *C*-terminal CBM consisting of three repeats of about 50 amino acids each (Martin-Cuadrado et al., 2008b). The deletion of this CBM termed SpEng1CBM caused a reduction of catalytic activity against insoluble substrates and a defect in targeting of Eng1p to the septum. No such CBM module has been found in other proteins of the GH81 family in *A. fumigatus, S. cerevisiae*, or *C. albicans*.

### Others exo-β-glucanases

**Figure 4:**
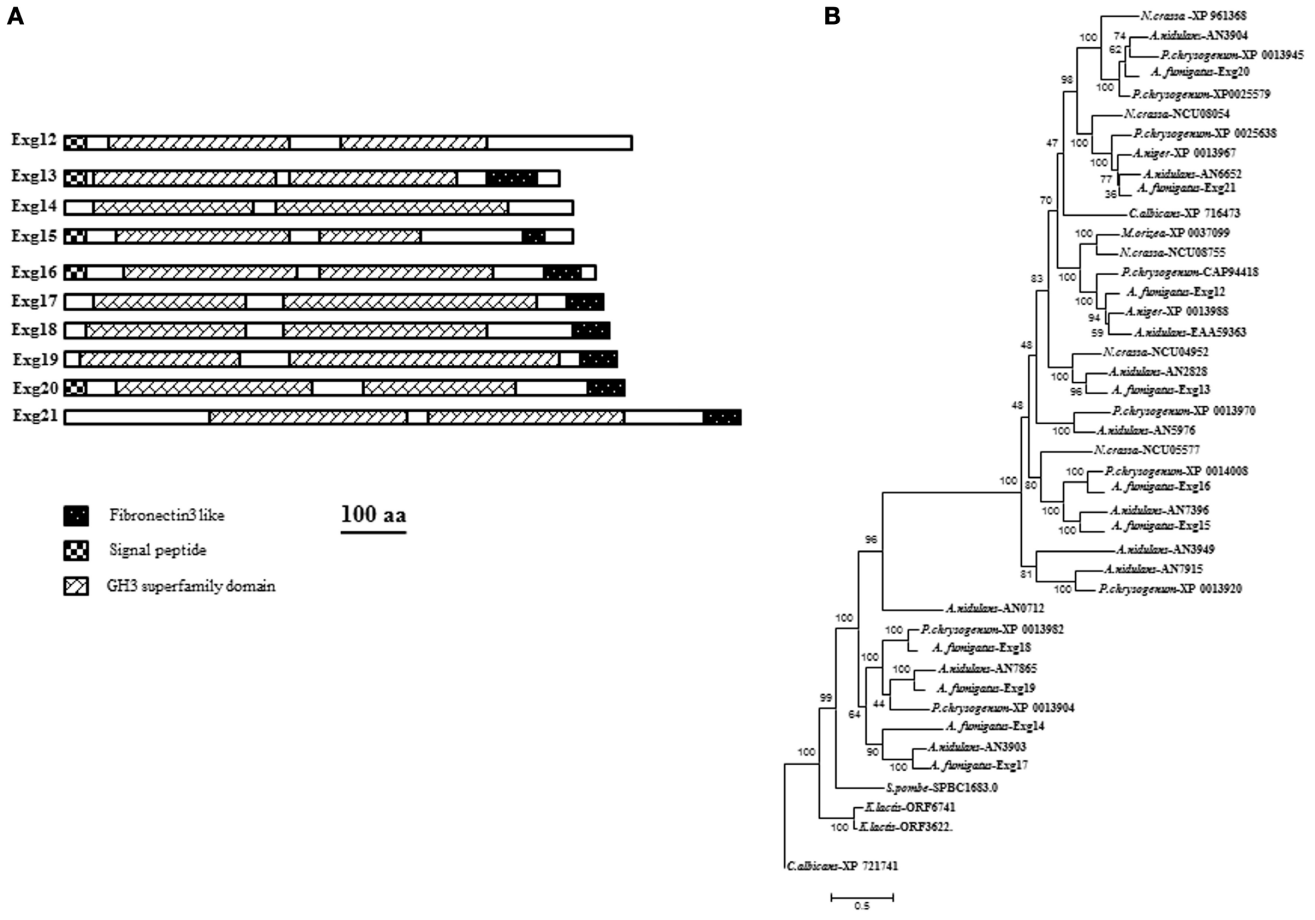
**(A)** Domain organization of GH3 families of *A. fumigatus*. Protein domains identified using SignalP 3.0, big-PI, TMHMM v. 2.0, and CD search (100 aa: protein length proportional to 100 amino acids). and **(B)** Phylogenetic tree of β(1–3)glucanases GH3 family.

In the CAZy database, family GH3 contains exo-hydrolases able to degrade different substrates. They are classified as β-glucosidase (EC 3.2.1.21); xylan 1,4-β-xylosidase (EC 3.2.1.37); β-N-acetylhexosaminidase (EC 3.2.1.52); glucan 1,3-β-glucosidase (EC 3.2.1.58); glucan 1,4-β-glucosidase (EC 3.2.1.74); exo-1,3-1,4-glucanase (EC 3.2.1.-); α-L-arabinofuranosidase (EC 3.2.1.55). Ten members of GH3 are present in the genome of *A. fumigatus* (Table 1): AFUA_1G05770, AFUA_7G06140, AFUA_7G00240, AFUA_1G17410, AFUA_8G02100, AFUA_6G14490, AFUA_5G07190, AFUA_6G11910, AFUA_6G08700 and AFUA_6G03570. The characteristics of the proteins encoded by these genes are summarized in Figure 4A and Table 1. Half of the proteins are secreted. All the proteins of *A. fumigatus* share conserved amino acids residues corresponding to the GH3 domain of the protein. Eight proteins have a Fibronectin like domain3 at the N-terminus. Fibronectin (FN) is a multidomain protein with the ability to bind simultaneously to cell surface receptors, collagen, proteoglycans, and other FN molecules (Schwarzbauer and De Simone, 2011). The phylogenetic tree is described in Figure 4B. Two main groups are observed. For most of the filamentous fungus, we observed a high redundancy of gene (from 5 to 11) in contrast to *C. albicans* (2) and *S. pombe* (only one). No orthologs have been found in the *S. cerevisiae* genome. To date, the only enzymatic activity characterized for this family in yeast or filamentous fungus correspond to ExoGII encoded by AFUA_1G05770 of *A. fumigatus* (Fontaine et al., 1997a). ExoGII has been isolated from the cell wall autolysate and is a 230 kDa protein able to degrade β-1,3-glucan as well as β-1,6-glucan and p-nitrophenyl-glucose (pNP-Glc). Such hydrolytic activity is in agreement with the classification of this enzyme in the GH3 family.

## What morphogenetic role plays the β(1,3)-glucanosyltransferase?

For many years, glucanosyltransferases were classified as glycosylhydrolases because their transglycosidase function was unknown. These glucanosyltransferases have indeed a glucanase activity because the first step in their sequential activity is to cleave linear β-1,3-glucans before the structural modification of the glucan (branching or elongation) in the second step. Because of their action on already established β-1,3-glucans, these enzymes could play a role in the structural modification of the cell wall and favor the emergence of a germ tube or an accessory hypha. Such roles have been suggested but never demonstrated.

### Branching enzyme

A glucanosyltransferase activity encoded by the *BGT1* gene has been identified biochemically in the *A. fumigatus* cell wall autolysate, Bgt1p belongs to the Carbohydrate-Active Enzyme (CAZy) database Glycosyl hydrolase GH17 (http://www.cazy.org/) (Table 1). Bgt1p cleaves a laminaribiose from the reducing end of linear β-(1,3)-glucans and transfers the remaining glucan to **the end** of another β-1,3-glucan acceptor with a β-(1,6)-linkage (Mouyna et al., 1998). Such activity had been described originally in *S. cerevisiae* (Klebl and Tanner, 1989). We recently characterized Bgt2p in *A. fumigatus*. This protein presents homology with Bgt1p (Gastebois et al., 2010a). Using a recombinant protein produced in *P. pastoris*, we showed that the enzymatic activity of Bgt2p is a new branching activity, able to cleave two residues of a β-1,3-glucan chain and transfer them to the **inside** of another chain of β-1,3-glucan with β-1,6-linkage. However, the single and double *bgt1/bgt2* mutants do not display a phenotype distinct from the parental strain (Mouyna et al., 1998; Gastebois et al., 2010a). Moreover, the amount of the β-1,3-glucan branching point has not changed in these mutants in comparison to the parental strain. Three other orthologs (AFUA_6G12380, AFUA_8G05610 and AFUA_5G08780) belonging to the GH17 family are currently investigated for their role as branching enzymes in the *A. fumigatus* cell wall.

### Elongation enzyme

Elongation of β-1,3-glucan is performed through the action of a β-1,3-glucanosyltransferase, which were originally discovered in *A. fumigatus*. This enzyme Gel1p splits a β-1,3-glucan molecule internally and transfers the newly generated reducing end to the non-reducing end of another β-1,3-glucan molecule, resulting in the elongation of the glucan chain (Hartland et al., 1996). Gel1p belongs to a family of seven members specifically recognized as the unique GH72 family in the Carbohydrate-Active Enzyme (CAZy) database (http://www.cazy.org/) (Mouyna et al., 2000a) (Table 1). This enzyme activity has been confirmed among paralogs of Gelp in *A. fumigatus* (Mouyna et al., 2000b; Gastebois et al., 2010b) and orthologs in other species like the *GAS* family in *S. cerevisiae* (Vai et al., 1991; Ragni et al., 2007), the *GAS* family in *S*. *pombe* (De Medina-Redondo et al., 2008, 2010) and *PHR* family in *C. albicans* (Saporito-Irwin et al., 1995; Muhlschlegel and Fonzi, 1997).

This GH72 family is subdivided in two families corresponding to the presence or absence of a Carbohydrate Binding Domain (CBM43) at the C-terminus. The Gel-family proteins are glycosylated and attached to the plasma membrane through a glycosylphosphatidylinositol (GPI) anchor. The recent crystallization of Gas2p, a Gelp homolog in *S. cerevisiae*, allows a better understanding of the enzyme's activity (Hurtado-Guerrero et al., 2009). Gas2p is composed of two interacting domains, a β(α)8 catalytic domain and a cystein-rich domain of the CBM43 family. The catalytic domain contains three disulfide bridges that are involved in the formation of the acceptor-saccharide binding site. The active site is defined by two catalytic residues, Glu176 and Glu275, and three tyrosine residues, Tyr107, Tyr244 and Tyr307, all conserved in the GH72 family. Moreover, although that seven homologs are present in the *A. fumigatus* genome, only *GEL1, GEL2*, and *GEL4* are expressed at each stage of growth in the conditions tested (Gastebois et al., 2010b). Deletion of each gene has a different effect on the phenotype. The *gel1* mutant behaves like the wild type, in contrast to the *gel2* mutant which showed reduced growth with several phenotypical changes and cell wall modification (Mouyna et al., 2005). In addition, it was shown that *GEL4* is an essential gene (Gastebois et al., 2010b). The morphogenetic role of these transglycosidases does not seem, however, associated to their glucanase function since these activity continue until the formation of an insoluble β-1,3-glucan.

### Cross-linking enzyme?

The *CRH* family proteins which are classified in GH16 were originally studied in *S. cerevisiae* and they have been suggested biochemically to be involved in the linkage between β-1,6-glucan and chitin (Cabib et al., 2007, 2008). Although there is no β-1,6-glucan in *A. fumigatus* cell wall, 5 *CRH* orthologous genes have been identified in the *A. fumigatus* genome, AFUA_6G03230, AFUA_2G03120, AFUA_3G09250, AFUA_6G08510 AFUA_1G16190, and belonging to the GH16_GPI_glucanosyltransferase subfamily (Table 1). Their biochemical function remains unknown in this fungus. In addition, single deletion of these genes was not associated to any growth phenotype modification (Chabane S, Reichard U, unpublished). Their role in morphogenesis will be only understood when the quintuple *crh* mutant will be obtained.

## Conclusions

Our understanding of the biochemical events responsible for conidial germination or hyphal branching remains incomplete. This review summarized the β-1,3-glucan modifying enzymes which have been described and the putative β-glucanases identified in *A. fumigatus* genome. Although the role of glycosylhydrolases during morphogenetic events has been suggested in filamentous fungus for many years, there is still no real proof of their importance during cell wall softening. The corresponding β-glucanases mutants obtained did not show any morphological differences compared to the wild type. In addition, if modifications of the glucanase activity have been seen in these mutants, the quantification of the activity is always performed *in toto* and never takes into account the localization *in situ* and their degrading capacity at a very specific cellular site. Such importance of the localization of the glycosylhydrolases has been already demonstrated in *S. pombe* (Martin-Cuadrado et al., 2008b). The redundancy of the putative exo β-or endo-glucanase in the genome (at least 29) make their analysis difficult because of putative compensatory mechanisms. The expression level of each of these putative β-glucanase during germination by RNA seq analysis is undergoing. Other hydrolases like chitinases that can have also similar function as glucanases will be jointly investigated. It should could give us some clue in the future to focus and study the genes upregulated during these morphogenesis events.

### Conflict of interest statement

The authors declare that the research was conducted in the absence of any commercial or financial relationships that could be construed as a potential conflict of interest.
